# Supplementary material for: Does Adding Standard Systematic Biopsy to Targeted Prostate Biopsy in PI-RADS 3 to 5 Lesions Enhance the Detection of Clinically Significant Prostate Cancer? Should All Patients with PI-RADS 3 Undergo Targeted Biopsy?
Source: Diagnostics (Basel). 2021 Jul 26;11(8):1335. doi: 10.3390/diagnostics11081335 (PMC8392157; doi:10.3390/diagnostics11081335)
Supplement: Supplementary file 1 [file diagnostics-11-01335-s001.zip › diagnostics-1284298-supplementary.pdf]

Supplemental Materials

Supplementary material and methods.

Supplemental mp-MRI protocol and characteristics

T1WI were acquired in the axial plane (TR 505 ms, TE 10 ms, field of view (FOV) 32.0 cm) to include the entire pelvis for the detection of enlarged pelvic lymph nodes and bone metastases, and for detection of post-biopsy hemorrhage. Slice thickness: 3 mm. Image matrix: 192 x 256. T2WI sequences were acquired in the axial and coronal planes. Axial plane: TR 4350 ms; TE 99 ms; FOV 20.0 cm; image matrix 310 × 320; slice thickness 3 mm. Coronal plane: TR 4000 ms; TE 99 ms; FOV 20.0 cm; image matrix 310 × 320; slice thickness 3 mm. T2-weighted 3D sequences were acquired in the sagittal plane (TR 1600 ms, TE 94 ms, FOV 16.0 cm, image matrix 248 × 256, slice thickness 1 mm). We performed DWI with three b values: 50; 400; and 800 s/mm2. Also, a 1400 b value (calculated) was used (TR 3800 ms, TE 73 ms, FOV 26.0 cm, slice thickness 3.5 mm, image matrix 114 × 114). Apparent diffusion coefficient maps were calculated by using the standard mono-exponential model. DCE required intravenous injection of gadolinium (Gadobutrol: Gadovist®, Bayer Pharma, Germany), 0.1 mmol/kg body weight (equivalent to 0.1 mL Gadovist 1.0 mmol/kg body weight). The rate of infusion was 3 mL/s. An acquisition of dynamic T1WI over the entire gland was performed (TR 4.80 ms, TE 2.17 ms, FOV 22.0 cm, slice thickness 2 mm, image matrix 179 × 256). Perfusion curves were generated with software for prostate MR reading (Syngo.via, MR prostate, Siemens AG, Erlangen, Germany) on the workstation. The duration of the whole mp-MRI examination was 31 minutes per patient.

VNav prostate fusion with MRI step by step.

Examinations were performed by using a 1.5-T system Siemens, Erlangen, Germany. All patients underwent MR imaging before biopsy. The biopsy was performed using a LOGIQ E9 XDCLEAR ultrasound (US) system (Milwaukee, USA) equipped with an end-fire endorectal biopsy probe (IC5-9 D), with an operating bandwidth of 9–5 MHz.

In the fusion US/MRI prostate biopsy, a prostate MRI is performed before biopsy and then, at the time of biopsy, the MRI images are fused to the ultrasound images to guide the urologist to the targets.

VNav (fusion MRI and US)

The patient was placed in routine transrectal US biopsy position and an electromagnetic transmitter is placed in a suitable position less than 50 cm from the probe. After activating VNav MRI data images were imported from PACS and the MR imaging series to be fused are selected and displayed and the targets on the MR images are identified. The MR imaging and US volumes are registered by selecting a reference plane in the lowest part of the gland (the apex, where MR imaging and US sections match the same plane) or others reference points, such as the bladder-neck, the urethra at the prostate apex, or the insertion of the seminal vesicle. The lesion can also be used for registration, as well as the use of planar adjustment of the urethra seen at MRI with that seen at US. Real-time freehand navigation and biopsy with live US image control can then be performed.

Table S1. Results of targeted biopsy according to the PI-RADS score.

PI-RADS v.2	n	csPCa	No-csPCa	No PCa
1	1	0 (0)	0(0)	1(100)
2	49	2(4.1)	5 (10.2)	42(85.7)
3	102	14(13.7)	5(4.9)	83(81.4)
4	245	134(54.7)	16(6.5)	95(38.8)
5	86	79(91.9)	0(0)	7(8.1)
